# Synchronous Ipsilateral Adrenal and Retroperitoneal Ganglioneuroma: A Unique Case of Large Tumor Mass

**DOI:** 10.1155/carm/7440806

**Published:** 2025-03-25

**Authors:** Abdolreza Mohammadi, Fardin Asgari, Ehsan Zemanati Yar, Mina Rezayat, Mahdi Najarzadegan, Seyed Mohammad Kazem Aghamir

**Affiliations:** Urology Research Center, Tehran University of Medical Sciences, Tehran, Iran

**Keywords:** adrenal mass, ganglioneuroma, retroperitoneal mass, synchronous tumors

## Abstract

This case report presents a rare instance of synchronous adrenal ganglioneuroma and an ipsilateral retroperitoneal mass in a 51-year-old woman with episodic hypertensive crises and increased symptom frequency. Imaging revealed a hypodense mass near the right kidney and an additional adrenal mass, which led to surgical intervention. Laboratory findings indicated hypercortisolism, and both masses were surgically removed by the laparoscopy approach. Postoperative pathology identified both masses as maturing-type ganglioneuroma, with no signs of malignancy or complications. This case highlights the diagnostic and therapeutic challenges of such rare presentations and emphasizes the importance of detailed imaging, surgical excision, and histopathological analysis. The potential link between elevated cortisol levels and ganglioneuroma, as observed here, underscores the need for further research into these benign tumors.

## 1. Introduction

Adrenal masses are often discovered incidentally, posing diagnostic challenges due to their varied origins, from benign lesions such as cysts, lipomas, and ganglioneuroma to malignant tumors. Ganglioneuroma, a rare benign tumor derived from neural crest cells, accounts for only 0.3%–2% of cases [1, 2]. Although benign, these tumors frequently mimic other adrenal masses, often requiring surgical intervention for a definitive diagnosis [3–6], especially when coexisting with tumors in nearby organs such as the kidney. Recognizing ganglioneuromas is critical since they can resemble more aggressive tumors, risking either overtreatment or undertreatment if misdiagnosed [1, 7]. Despite advances in imaging technologies such as CT and MRI, distinguishing ganglioneuroma remains difficult. Indicators such as homogeneous appearance, calcifications, and weak contrast enhancement may point to ganglioneuroma but are not definitive [2, 8]. Consequently, surgical removal is typically needed, particularly for larger or atypical tumors, as malignancy cannot be excluded solely through imaging [1, 4]. The coexistence of a ganglioneuroma in the adrenal gland with another mass in the ipsilateral kidney is extremely rare, and few cases have been reported. This rarity presents important clinical considerations about the possible shared causes, the optimal surgical approach for managing both tumors and long-term patient outcomes and monitoring.

## 2. Case Presentation

**Figure 1:**
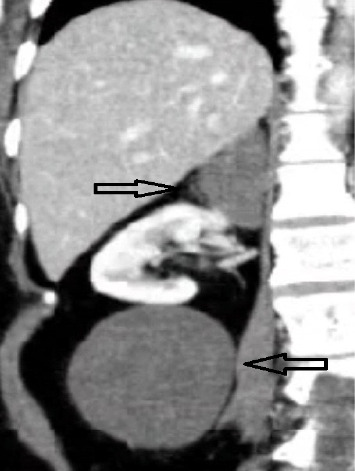
The upper arrow indicates a well-defined adrenal mass in the right adrenal gland. The lower arrow shows a hypodense retroperitoneal mass near the lower pole of the right kidney.

This case is reported based on CARE guidelines, and the patient agreed to report his case by signing the written informed consent. A 51-year-old woman, with a known case of episodic hypertensive crises and episodes of hot flashes on and off for approximately one year, presented to our clinic for further evaluation due to an increase in symptom frequency. Physical examination was normal, and there was no past medical history but hypertension that responded to medical therapy and without past surgical history and no history of smoke and alcohol. An incidental ultrasonography found a large hypodense mass near the lower pole of the right kidney. This warranted further workup with contrast-enhanced computed tomography (CT), which revealed a hypodense mass adhesion in the lower pole of the right kidney with an additional mass in the right adrenal gland (Figure 1).

Laboratory evaluation before surgery revealed key findings: elevated 24 h urine-free cortisol levels at 105.0 μg/24 h (normal range: 1.5–63 μg/24 h), suggesting hypercortisolism, while the patient's serum morning cortisol was low at 1.00 μg/dL (normal range: 3.7–19.4 μg/dL), indicating possible adrenal suppression or HPA axis disorder. Urine metanephros, normetanephrine, renin, and aldosterone levels were normal, ruling out pheochromocytoma or primary aldosterone as causes of hypertensive episodes. Other routine lab tests were normal.

**Figure 2:**
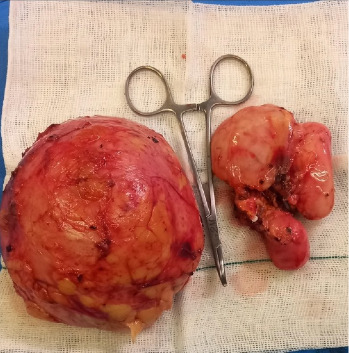
Intraoperative gross specimen showing the retroperitoneal mass (left; 10.1 cm × 8.3 cm) and the adrenal mass (right; 6.5 cm × 3.8 cm), both excised intact without rupture.

Due to complex lab findings, surgery was decided. Prophylactic antibiotics and intestinal preparation were given to reduce infection and contamination risks. During surgery, the patient was positioned in the right flank under general anesthesia. A 10-mm trocar was inserted to establish pneumoperitoneum, followed by three more trocars. The colon was mediatized, and the liver was mobilized to access the right adrenal mass, which was carefully dissected from the inferior vena cava (IVC), liver, and kidney. After ligating the adrenal vein, the adrenal mass was removed en bloc. Attention then shifted to a second retroperitoneal mass near the lower pole of the right kidney, which was dissected and removed intact without rupture. Both masses were extracted through a McBurney incision (Figure 2). There was no post-op complication, and the patient was discharged after 3 days.

Three weeks later, the pathological examination of the excised tissues revealed important findings. The histopathological analysis confirmed the retroperitoneal mass as a maturing-type ganglioneuroma, measuring 10.1 × 8.3 cm. The tumor was well-circumscribed, composed predominantly of spindle-shaped, wavy, and uniform Schwannian cells forming an organized stroma. Mature and immature ganglion cells were dispersed throughout the stroma, characterized by eccentric nuclei and abundant cytoplasm. The background displayed evident myxoid changes, enhancing the diagnostic features of ganglioneuroma. No evidence of atypia, mitosis, or necrosis was observed, and muscle-like bundles were interspersed within the tumor matrix.

**Figure 3:**
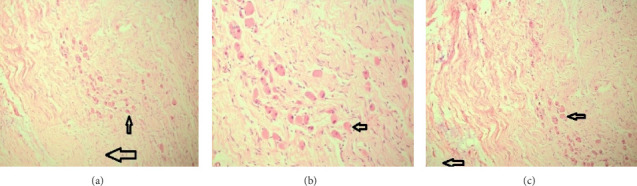
(a) Histological section showing Schwannian stroma (larger arrow) with organized structure and ganglion cells (smaller arrow) having eccentric nuclei. (b) Ganglion cells with eccentric nuclei (arrow) dispersed within the stroma, accompanied by evident myxoid changes. (c) Schwannian stroma (larger arrow) with ganglion cells (smaller arrow) exhibiting eccentric nuclei. Myxoid changes (bottom and left arrows) are visible, with no signs of atypia, mitosis, or necrosis.

Similarly, the adrenal mass was identified as a maturing-type ganglioneuroma, measuring 6.5 × 3.8 cm. This mass shared identical histological characteristics, with a well-defined border and an intact rim of normal adrenal tissue at the periphery. The surgical margins for both masses were negative for tumor cells, confirming complete excision and the benign nature of these neoplasms (Figures 3(a), 3(b), 3(c)).

## 3. Discussion

This case report presents the rare occurrence of a synchronous adrenal ganglioneuroma and an ipsilateral retroperitoneal mass, illustrating the diagnostic and therapeutic challenges associated with such presentations. Advanced imaging techniques such as CT and MRI were essential in identifying and characterizing the masses before surgery. Surgical removal and detailed histopathological examination confirmed the diagnosis and ensured complete excision, which is crucial for patient outcomes.

“Histopathology revealed no significant adrenal cortical changes to explain elevated cortisol. While this case offers insights into managing rare synchronous tumors, the lack of postoperative cortisol follow-up limits the long-term understanding. Future studies should incorporate follow-up data to explore endocrine implications.”

However, the rarity of this condition limits the generalizability of the findings. An interesting aspect of this case is the potential link between elevated cortisol levels and ganglioneuroma. While the case provides valuable insights, its uniqueness means that the findings may not apply broadly. Additionally, the lack of genetic or molecular analysis leaves unanswered questions about the underlying causes, and the absence of long-term follow-up data prevents the assessment of recurrence risk and long-term outcomes. Adrenal ganglioneuroma, benign tumors derived from neural crest cells, account for 0.3%–2% of adrenal tumors [1, 2]. These tumors are often discovered incidentally during imaging for unrelated conditions, as they are typically asymptomatic and associated with normal lab tests [3]. Differentiating ganglioneuroma from other adrenal masses, such as pheochromocytoma and adrenal cortical carcinomas, based on imaging alone is challenging [4, 9]. Common features include a homogeneous appearance, poor contrast enhancement, and calcifications, though these are not definitive [3]. The occurrence of synchronous adrenal and renal masses is extremely rare, with few cases reported. Surgical intervention was necessary to confirm the diagnosis and exclude malignancy, which is recommended when imaging cannot definitively characterize the masses [1]. Histopathological findings confirmed both tumors as benign ganglioneuroma, aligning with the known behavior of this nonfunctioning neoplasm.

## 4. Conclusion

This case emphasizes the importance of a comprehensive diagnostic approach for managing incidental adrenal and renal masses. Our reported synchronous ganglioneuroma is a rare case that presents diagnostic challenges that emphasized on detailed imaging, multidisciplinary surgery, and histopathological examination to differentiate them from malignant tumors and ensure proper patient management; the rise in cortisol may be one manifestation of these benign tumors.

## Data Availability

All data supporting the findings of this study are included within the manuscript and supporting information. However, more detailed data will be provided by the corresponding author on request.
